# Partitioning and integrating of plant traits and phylogeny in assessing diversity along secondary forest succession in Loess Plateau of China

**DOI:** 10.1002/ece3.10055

**Published:** 2023-05-10

**Authors:** Yongfu Chai, Shen Qiu, Kaiyue Wang, Jinshi Xu, Yaoxin Guo, Mao Wang, Ming Yue, Mingjie Wang, Jiangang Zhu

**Affiliations:** ^1^ Key Laboratory of Resource Biology and Biotechnology in Western China Northwest University Xi'an China; ^2^ School of Life Sciences Northwest University Xi'an China; ^3^ College of Grassland and Environment Sciences Xinjiang Agricultural University Urumchi China; ^4^ Shuanglong State‐owned Ecological Experimental Forest Station of Qiaoshan State‐owned Forestry Administration of Yan'an City Yan'an China

**Keywords:** community assembly, community diversity, forest succession, functional trait, phylogeny, trait–gradient analysis

## Abstract

Assessing plant diversity during community succession based on plant trait and phylogenetic features within a community (alpha scale) and among communities (beta scale) could improve our understanding of community succession mechanism. However, whether changes of community functional diversity at alpha and beta scale are structured by different traits and whether integrating plant traits and phylogeny can enhance the ability in detecting diversity pattern have not been studied in detail. Thirty plots representing different successional stages were established on the Loess Plateau of China and 15 functional traits were measured for all coexisting species. We first analyzed the functional alpha and beta diversity along succession by decomposing species trait into alpha and beta components and then integrated key traits with phylogenetic information to explore their roles in shaping species turnover during community succession. We found that functional alpha diversity increased along successional stages and was structured by morphological traits, while beta diversity decreased during succession and was more structured by stoichiometry traits. Phylogenetic alpha diversity showed congruent pattern with functional alpha diversity because of phylogenetic conservation of trait alpha components (variation within community), while beta diversity showed incongruent pattern due to phylogenetic randomness of trait beta components (variation among communities). Furthermore, only integrating relatively conserved traits (plant height and seed mass) and phylogenetic information can raise the detecting ability in assessing diversity change. Overall, our results reveal the increasing niche differentiation within community and functional convergence among communities with succession process, indicating the importance of matching traits with scale in studying community functional diversity and the asymmetry of traits and phylogeny in reflecting species ecological differences under long‐term selection pressures.

## Graphical abstract

Scale‐trait math of functional diversity shapes the increasing niche differentiation within community and functional convergence among communities with succession process. Long‐term selection pressure during forest succession leads the asymmetry of traits and phylogeny in reflecting species ecological differences.

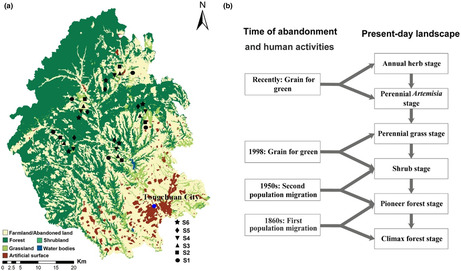

## 1 INTRODUCTION

There has been a paradigm shift in community ecology over the past two decades in how diversity is assessed and how diversity is linked with ecological mechanisms controlling community assembly (Cadotte et al., 2019; Dehling et al., 2022). The traditional quantification of species alpha and beta diversity can only provide relatively sparse information (Gong et al., 2019; Laughlin, 2014). In recent years, plant functional traits and phylogenetic status have contributed to assessing the biodiversity patterns by quantifying coexisting species differences using the evolutionary history or functional strategy, which improving our understanding of classic community assembly theory (De Pauw et al., 2021; Pavoine & Ricotta, 2014; Peres‐Neto et al., 2012; Shivaprakash et al., 2018). Although studies of phylogenetic diversity (PD) and functional diversity (FD) have provided valuable insights into community assembly, every approach has important assumptions and limitations, which affects their usefulness and interpretability (Cadotte et al., 2013; Gianuca et al., 2016; Gong et al., 2019; Laughlin, 2014; Sena et al., 2018).

Based on the trait‐based framework, species niche difference is quantified as a distance among pairwise species within a multidimensional trait space (Spasojevic et al., 2014). Nevertheless, we could only consider a limited trait number in the actual studies, thus the selection of informative traits and their statistical or mechanistic link with ecological processes need to be considered (Butterfield & Suding, 2013). In theory, assembly processes act hierarchically on different scales (within community or among communities; Götzenberger et al., 2012), which leads to different variations of traits within a community and among communities (Gianuca et al., 2016). Therefore, mismatches between trait and scale may skew our ability to detect assembly pattern (Gianuca et al., 2016; Messier et al., 2010). Trait–gradient analysis is a trait‐based framework to complete trait–scale matches that provides a guideline to select traits (Ackerly et al., 2006; Ackerly & Cornwell, 2007). Using this framework, species trait values can be decomposed into alpha and beta components: trait alpha components refer to the within‐community dimension of niche differentiation, while beta trait components refer to the among communities dimension of niche differentiation (Cavender‐Bares et al., 2009; Silvertown et al., 2006). Trait–gradient analysis may offer the possibility of detecting trait‐based niche differentiation within community and among communities.

In addition, the use of phylogeny in ecology is based on the assumption that closely related species are more similar than distantly related species (Cadotte et al., 2017; Gianuca et al., 2016). However, a potential limitation is that traits can be plastic, so their convergence and divergence along phylogenies may vary according to trait's plasticity (Sena et al., 2018). Therefore, the phylogeny and traits of species may reflect the ecological difference in different ways. If phylogeny provides necessary complemental information other than that by the measured traits (Cavender‐Bares et al., 2009; Mouquet et al., 2012), information provided by phylogeny and traits can be integrated to enhance community assembly inference. The advantage of integrating multiple traits and phylogeny in assessing diversity pattern has been tested in alpine plant communities along an elevation gradient (Cadotte et al., 2013). If functional diversity is structured by different traits within a community and among communities, thus integrating specific traits with phylogeny at different scales is helpful for understanding diversity pattern along environmental gradient. This hypothesis has been confirmed in assessing functional diversity of zooplankton in farmland ponds (Gianuca et al., 2016), while this has not been tested in plant communities.

Secondary forest succession can be regarded as a re‐assembled process of communities following natural or anthropogenic disturbance (Prach & Walker, 2011). It includes multiple community assembly processes such as dispersal limitation, species pool effects, priority effects, abiotic environmental filtering, stochastic process, and biotic interactions (Chang & Turner, 2019; Li et al., 2015; Lohbeck et al., 2014). A given forest secondary succession series usually exhibits a predictable species turnover as characteristic species compositions emerge at a given stages of community succession (Bruelheide et al., 2011; Chai et al., 2016; Måren et al., 2017). Replacement of species could lead to changes of physiological and functional structure within and among community because of abiotic environmental filtering or biotic interactions. Some studies have separately compared the change in functional trait and phylogenetic diversity during community succession (Baraloto et al., 2012; Chua & Potts, 2018; Shooner et al., 2015). However, it is not clear whether integration of traits and phylogenetic information is meaningful in understanding community diversity during forest secondary succession, although such succession series are highly suitable systems for studying species diversity maintenance during vegetation restoration (Bruelheide et al., 2011).

**FIGURE 1:**
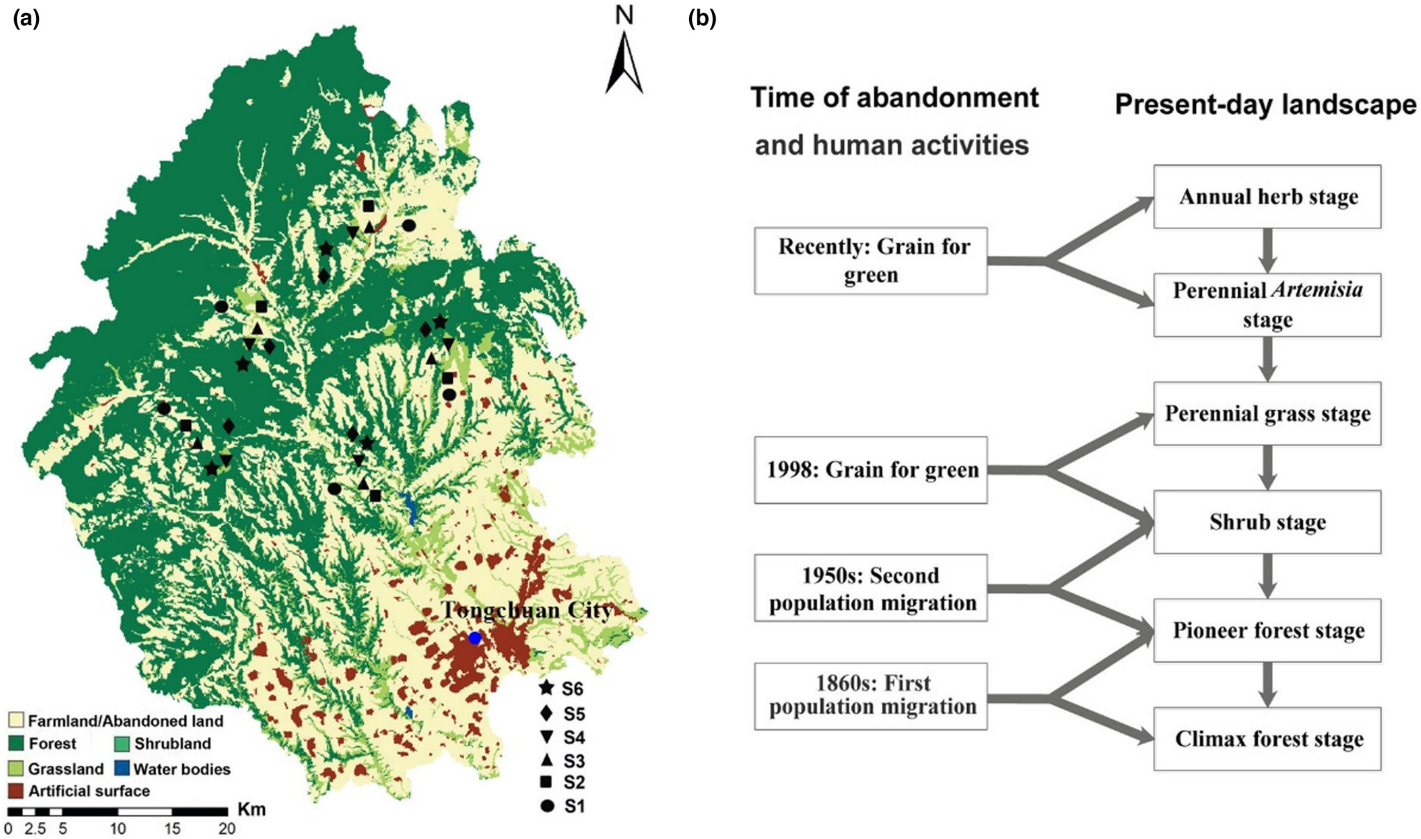
Plot distribution and successional model of the community following abandoned agricultural fields on the Loess Plateau of China. The land‐cover map of the study area on the Loess Plateau is based on normalized‐difference vegetation index in 2015 (a). Diagram of successional model (b) is modified from Chai et al. (2019). S1–S6 represent different successional stage after abandonment, respectively.

The Loess Plateau in northern China, a semi‐arid ecosystem, suffers from deep loess and severely depleted soils and water (Kou et al., 2016; Wang et al., 2018). On the plateau, most plant communities are transitioning from abandoned agriculture to forest climax communities reflecting a time series of anthropogenic disturbances since the 1860s (Chai et al., 2015, 2016; Zhu, 1993) (Figure 1). It is rare to explore forest succession in a semi‐arid ecosystem from a functional and phylogenetic perspective. In the present study, 15 plant functional traits were selected to explore scale‐traits match and understand the roles of plant functional trait and phylogenetic distance in determining plant community diversity during a long‐term (≥150 years) succession from arable land to climax forest community on the Loess Plateau. The selected traits were representative of plant resource capture, competition, or defense strategies (Table S1) (Laughlin, 2014; McGill et al., 2006; Navas et al., 2010; Violle et al., 2009; Wright et al., 2005). The study aims to explore three questions (1) Is functional diversity during forest succession structured by specific traits at different scales (i.e., alpha scale within community; beta scale among communities). Can decomposition of these traits improve the explanatory power of successional ages on functional diversity? (2) If traits have strong phylogenetic signal (e.g., conserved traits along phylogeny), do traits and phylogeny support congruent diversity pattern during forest succession? (3) Are there some appropriate traits that can be integrated with phylogenetic information to raise the detecting ability of diversity pattern during forest succession?

## 2 MATERIALS AND METHODS

### 2.1 Study site

This study was conducted in the Ziwuling reserve region (35°09′‐35°40′ N, 108°47′‐108°57′ E) of the Loess Plateau, Shaanxi, China (Zhu, 1993). There is a mean annual precipitation of 550–650 mm, which is mainly concentrated in the summer. Mean annual temperature ranges from 9 to 11°C, and elevation ranges from 1100 to 1150 m.

Natural vegetation in this region ever undergone anthropogenic forest disturbance in the form of logging or clearing for agriculture. Population migrations and the Grain for Green program led secondary succession to begin on abandoned field, and similar history of land use, regional species pool and climate condition result in that community succession progresses along a similar successional trajectory toward secondary climax forests (Figure 1). All community fragments in the present‐day landscape can be classified into six successional stages, annual herb stage, perennial *Artemisia* stage, perennial grass stage, shrub stage, pioneer forest stage, climax forest stage (Chai et al., 2016, 2019; Zhu, 1993; Figure 1). These stages can be identified by the community species composition and the time of field abandonment with the help of local chronicles and previous surveys.

According to our previous investigation of the vegetation in the study area, we firstly selected five sites in the study area, each site includes different communities representing six successional stages. In order to avoid spatial autocorrelation and pseudo‐replication, distances between sites exceeded at least 1 km. Six plots (20 m × 20 m) representing six succession stages were established within each site. Finally, a total of 30 plots (5 plots per stage × 6 stages) were established. These plots were established in 2013 and were surveyed in 2014. The secondary successional stage has an age representing a time series as: Stage one (1 year) was dominated by annuals, whereas stage two (3 years) was dominated by herbaceous perennials *Artemisia gmelinii* and *Artemisia lavandulaefolia* (Compositae). Perennial grasses (Gramineae) dominated stage three (15 years), and stage four (40 years) was dominated by shrub community. The dominant growth form in stage five was pioneer tree species (100 years). *Quercus* species dominated the climax forest stage (150 years). The successional age was determined based on official records for herb community (stage 1 to stage 3) and tree core analysis was used for shrub and forest community (stage 4 to stage 6).

### 2.2 Functional traits and community phylogeny

In all plots, 210 vascular plant species belonging to 59 families and 162 genera were identified (Table S2), and a minimum of five individuals were sampled for trait measurements within each plot. For the woody species, completely developed sun‐exposed leaves were randomly sampled from five healthy branches at a rate of approximately 10–20 leaves per individual. The stem and root of the same individual were sampled; roots were sampled by excavating the first 20–30 cm of the soil depth near the plant basal stem. For the herbaceous species, leaves, stems, and roots were sampled from five random 0.5 × 0.5 m quadrats within each plot.

Fifteen traits from different organs were selected to indicate multidimensional functions of plants including resource use, dispersal, and competitive ability (Table S1). Measurements of functional traits were generally conducted according to the protocols of Cornelissen et al. (2003) and Chai et al. (2015). Fresh mass (*M*_F_) of leaves, stems and roots were weighted by an electronic balance (±0.0001 g) and leaves were photographed immediately using a digital camera. Then, dry mass (*M*_D_) was measured after all samples were dried in an oven for 72 h at 80°C. Leaf areas (LA) were measured with motic images plus 2.0 software (Motic China Group Co., Ltd.). Then specific leaf area (SLA) was calculated as LA/*M*_D_. Leaf dry mass content (LDMC), stem dry mass content (SDMC), and root dry mass content (RDMC) were calculated as *M*_D_/*M*_F_. An elemental analyzer (Euro Vector EA3000) was used to determine the leaf carbon content (LCC), leaf nitrogen content (LNC), stem carbon content (SCC), stem nitrogen content (SNC), root carbon content (RCC), and root nitrogen content (RNC). Following digestion of H_2_SO_4_–H_2_O_2_, leaf phosphorus content (LPC) was determined by using ammonium molybdate spectrophotometric method (Bowman, 1988). Plant height (Height) was measured as the distance between the top of the photosynthetic tissues of each individual and the soil surface. Seeds mass (SM) of most species were collected from field. For 19 species that the number of seeds was insufficient, the mean of congeneric species was used as the estimate.

The informatics tool phylomatic (http://www.phylodiversity.net) was used to construct a phylogeny of all 210 species (Webb & Donoghue, 2005). The Angiosperm Phylogeny Group's APGIII consensus tree (Zanne et al., 2014) was used as a backbone, then species were added onto it based on their taxonomy. After branch lengths were obtained by the BLADJ algorithm (Webb et al., 2008), a phylogenetic distance matrix (PDist) was constituted.

### 2.3 Data analyses

#### 2.3.1 Partitioning of species mean trait values and estimates of phylogenetic signal in traits

All traits data were transformed to a log scale before analysis. We calculated the community‐weighted mean trait (*p*_*j*_; Equation 1) and species mean trait value (*t*_*i*_; Equation 2; Ackerly & Cornwell, 2007). The community weighted mean trait and species mean trait values were further partitioned into beta (*β*_*i*_; Equation 3) and alpha (*α*_*i*_; Equation 4) components by using trait–gradient analysis (Ackerly & Cornwell, 2007). Alpha trait components are the difference between a species trait values and the mean of co‐occurring species; beta trait components refer to a species position along a gradient defined by community‐level mean trait values (Ackerly & Cornwell, 2007). Different components were then integrated separately with species phylogenetic information (Cadotte et al., 2013). Phylogenetic signal of species mean trait values, beta trait components, and alpha trait components were investigated using the *K* statistic (Blomberg et al., 2003).

(1) $$p_{j}=\frac{\sum_{i=1}^{S}a_{\mathrm{ij}}\times t_{\mathrm{ij}}}{\sum_{i=1}^{S}a_{\mathrm{ij}}}$$

(2) $$t_{i}=\frac{\sum_{j=1}^{P}a_{\mathrm{ij}}\times t_{\mathrm{ij}}}{\sum_{j=1}^{P}a_{\mathrm{ij}}}$$

(3) $$\beta_{i}=\frac{\sum_{j=1}^{P}p_{j}\times t_{\mathrm{ij}}}{\sum_{j=1}^{P}a_{\mathrm{ij}}}$$

(4) $$\alpha_{i}=t_{i}-\beta_{i}$$

where *t*_*ij*_ and *a*_*ij*_ is the trait value and relative coverage of species *i* in plot *j*, and *S* and *P* is the total number of species and plots, respectively.

#### 2.3.2 Selection of traits in assessing functional diversity at different scales

First, the distribution of the data for each trait of all species was centered, and an Euclidean distance matrix was calculated. We then calculated the mean functional distance (FDist) between all pairs of species co‐occurring for each plot as the functional alpha diversity. Beta functional diversity was estimated using the mean pairwise functional distance among pairs of communities within each successional stage, which is performed by the function COMDIST in the Picante statistical package for r software (Kembel et al., 2010). All functional diversity metrics were calculated based on the species mean trait values, alpha and beta trait values, respectively.

The Pearson correlations between functional diversity and succession age were analyzed to select the informative traits at different scales. Trait distances that were significantly correlated with successional age were selected as the informative traits at the corresponding scale. We also compared the difference of correlation coefficients among different trait components using the “cocor” package in the r software (version 4.1.0; R Development Core Team, 2020) to test the effectiveness of trait–gradient analysis (Diedenhofen & Musch, 2015). Using the data of informative traits, linear or quadratic polynomial regression were then performed to determine the optimal changing pattern of functional diversity with successional stages. We also analyzed the correlations between functional diversity and soil condition heterogeneity. Soil condition heterogeneity was assessed by Euclidean distance of soil factors between two plots. Soil dataset is from Chai et al. (2019).

#### 2.3.3 Phylogenetic diversity at different scales

We used Faith's phylogenetic diversity (PD) metric to indicate the phylogenetic alpha diversity (Faith, 1992). Faith's PD calculated the mean phylogenetic distance (PDist) between all pairs of species co‐occurring within a community, it has been widely used in phylogenetic diversity research (Morlon et al., 2011). Phylogenetic beta diversity was calculated using the same method as functional beta diversity.

#### 2.3.4 Integration of phylogenetic and trait distances

We integrated selected informative traits components with phylogeny to calculate functional–phylogenetic diversity at different scales. Briefly, functional–phylogenetic distance matrices were constructed (FPDist) based on the phylogenetic and functional distance matrices after they scaled between 0 and 1 (Equation 5; Cadotte et al., 2013). The weighting parameter a‐value was used to adjust the relative importance of trait and evolutionary differences. At intervals of 0.01, we constructed 101 FPDist matrices corresponding to a‐values from 0 to 1. In the case of *a* = 0, FPDist represents pure functional trait distances, whereas in the case of *a* = 1, FPDist represents pure phylogenetic distances. An intermediate a‐value indicates that the two sets of information have been integrated.

(5) $$\text{FPDist}={\left(a{\text{PDist}}^{p}+\left(1-a\right){\text{FDist}}^{p}\right)}^{1/p}$$

where *p* represents the *p*‐norm distance. Euclidean distance was used in our study, thus we combined functional and phylogenetic distances with *p* = 2.

#### 2.3.5 Functional–phylogenetic diversity along forest succession

The FPDist representing functional–phylogenetic alpha diversity (alpha FPD) was calculated across a‐values at the alpha scale for all plots. Functional–phylogenetic beta diversity (beta FPD) was calculated across a‐values based on the mean pairwise functional–phylogenetic dissimilarity among pairs of communities within each successional stage (Cadotte et al., 2013). These metrics were performed by the function COMDIST of the picante package in r software (Kembel et al., 2010). We then assessed the relationship between diversity and successional age across a‐values, and coefficient of determination (*R* ^2^) was used as a measurement for goodness of fit (Cadotte et al., 2013). This also allowed us to find the fit a‐value that maximize the explanatory power (*R* ^2^). Both quadratic polynomial regression and linear regression were applied to obtain the most adequate model. All statistical analyses were performed with r software (version 4.1.0; R Development Core Team, 2020).

## 3 RESULTS

### 3.1 Phylogenetic signal of traits

The *K* statistic revealed that all trait components of plant height and SM show the strongest phylogenetic signal. For most of the other traits, alpha trait values showed weak (*K* < 1) but significant phylogenetic signal, while beta trait values did not show significant phylogenetic signal (Table 1, Figures S1–S3).

**TABLE 1:** Phylogenetic signal of different traits components. The explanations of trait acronyms can be found in Table S1.

Traits	Species trait		Alpha comp.		Beta comp.	
	*K*	*p*	*K*	*p*	*K*	*p*
Height	1.16	**.001**	0.63	**.001**	0.34	**.007**
SM	0.67	**.003**	0.52	**.002**	0.35	**.033**
SSD	0.27	.057	0.24	.234	0.26	.122
SDMC	0.47	**.001**	0.44	**.001**	0.24	.437
SCC	0.35	**.002**	0.27	.145	0.25	.425
SNC	0.40	**.001**	0.40	**.001**	0.27	.318
RDMC	0.37	**.001**	0.35	**.001**	0.21	.820
RCC	0.41	**.001**	0.35	**.001**	0.26	.165
RNC	0.38	**.001**	0.40	**.001**	0.23	.458
N:P	0.29	**.007**	0.36	**.001**	0.25	.133
LPC	0.31	**.025**	0.29	**.016**	0.27	.397
LDMC	0.44	**.001**	0.43	**.001**	0.21	.791
LCC	0.30	**.026**	0.31	**.027**	0.23	.523
LNC	0.36	**.001**	0.36	**.001**	0.24	.310
SLA	0.34	**.001**	0.40	**.001**	0.25	.223

*Note*: Bold represents *p* < .05.

### 3.2 Functional diversity along succession indicates trait–scale match

**FIGURE 2:**
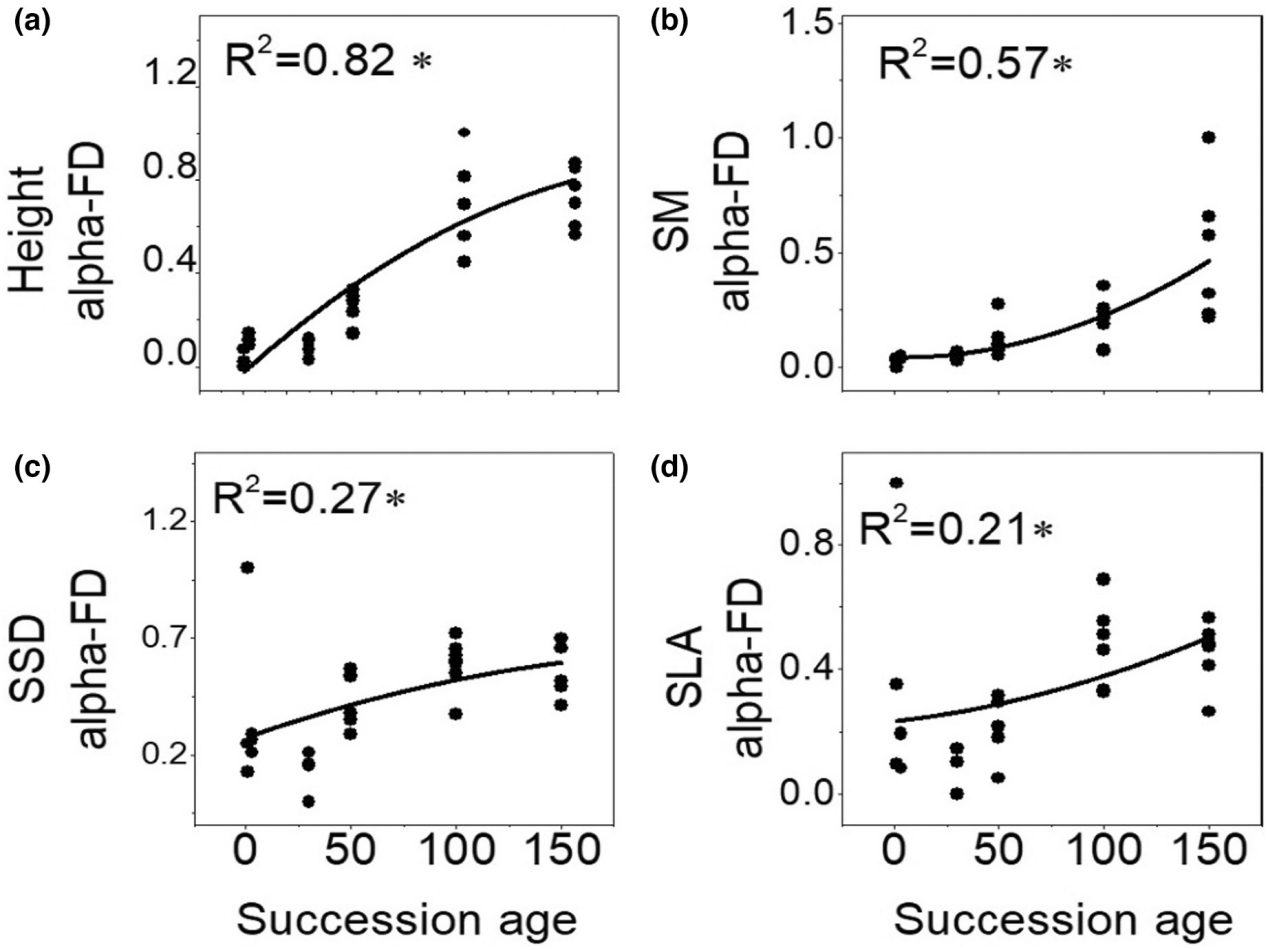
Quadratic polynomial regression for functional alpha diversity as dependent variable and successional age as the independent variable. According to preceding analyses (Table 2), alpha components of four traits (Height, SM, SSD, and SLA) were used for assessing alpha diversity. Height, plant height; SLA, specific leaf area; SM, seed mass; SSD, stem‐specific density.

**FIGURE 3:**
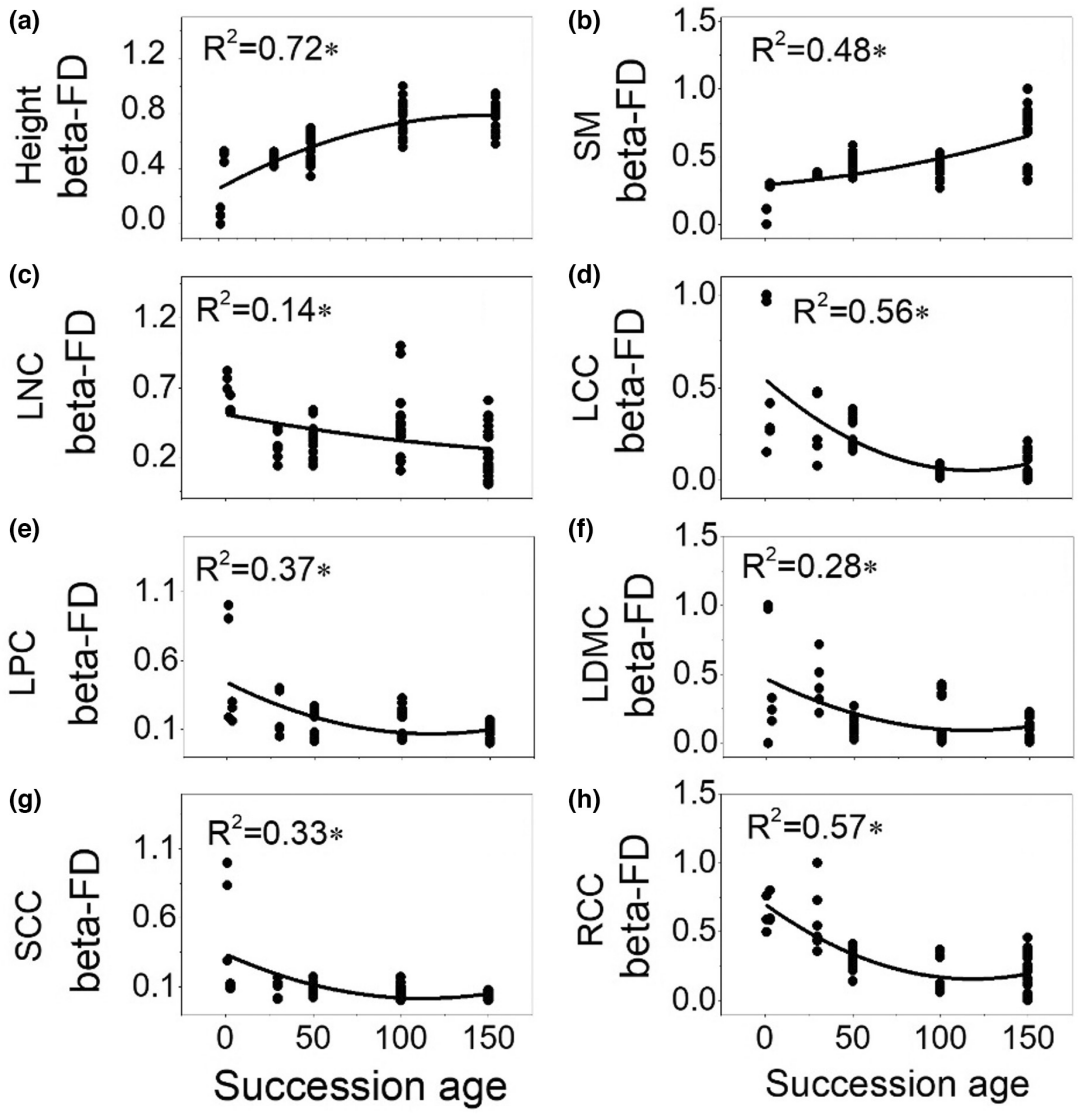
Quadratic polynomial regression for functional beta diversity as dependent variable and successional age as the independent variable. According to preceding analyses (Table 2), beta components of eight traits (Height, Seed mass, LNC, LCC, LPC, LDMC, SCC, RCC) were used for assessing beta diversity. Height, plant height; LCC, leaf carbon content; LDMC, leaf dry mass content; LNC, leaf nitrogen content; LPC, leaf phosphorus content; RCC, root carbon content; SCC, stem carbon content; SM, seed mass.

Whether linear regression or nonlinear regression, four morphologic traits (Height, SM, SSD, and SLA) were informative in assessing the change of functional alpha diversity along succession and eight traits (Height, SM, SCC, RCC, LPC, LDMC, LCC, LNC) were appropriate for assessing the change of functional beta diversity (Table S3). Functional alpha diversity increased significantly with successional age (Figure 2). Beta diversity of six stoichiometry traits (LCC, RCC, SCC, LDMC, LPC, LNC) declined significantly with successional age, while beta diversity of plant height and SM increased with successional age (Figure 3). On the contrary, using all traits in analyses of functional diversity was suboptimal compared with consideration of informative traits separately at a given scale (Table S3).

Specially, functional alpha diversity based on alpha trait values was more strongly correlated with successional age than that based on species mean traits or beta values (Table 2). Conversely, functional beta diversity based on beta trait values responded more strongly than species trait and alpha trait values to successional age (Table 2). Moreover, beta trait values were more correlated than alpha trait values, suggesting niche differentiation within communities and habitat filtering of trait in species turnover among communities (Figure S4).

**TABLE 2:** Using appropriate trait components enhance the correlation between functional diversity and succession age. Four traits for alpha scale and eight traits for beta scale were used according to pre‐analysis (see in Table S3).

Traits	Species trait	*α* comp.	*β* comp.	*p‐*Value
Functional alpha diversity				
Height	.87 (.76)	**.90 (.81)**	.75 (.56)	*
SM	.70 (.50)	**.73 (.53)**	.60 (.36)	*
SSD	.38 (.14)	**.51 (.26)**	.03 (.00)	*
SLA	.37 (.14)	**.45 (.20)**	−.05 (.00)	*
Functional beta diversity				
Height	.77 (.59)	.82 (.67)	**.85 (.72)**	NS
SM	.59 (.35)	.64 (.41)	**.69 (.48)**	NS
SCC	−.38 (.14)	−.16 (.03)	**−.46 (.21)**	*
RCC	−.58 (.33)	−.32 (.10)	**−.64 (.41)**	*
LPC	−.36 (.13)	−.33 (.11)	**−.50 (.25)**	*
LDMC	−.34 (.12)	−.17 (.03)	**−.44 (.19)**	*
LCC	−.56 (.31)	−.38 (.14)	**−.63 (.40)**	*
LNC	−.35 (.12)	−.12 (.01)	**−.37 (.14)**	*

*Note*: Correlation coefficient *r* out of parentheses and *R* ^2^ in parentheses. Asterisk indicates significant difference among correlation coefficients of *α* and *β* component. NS indicates no significant. The maximal *R* ^2^ for a certain trait is given in bold.

### 3.3 Phylogenetic diversity along succession and its integration with traits

**FIGURE 4:**
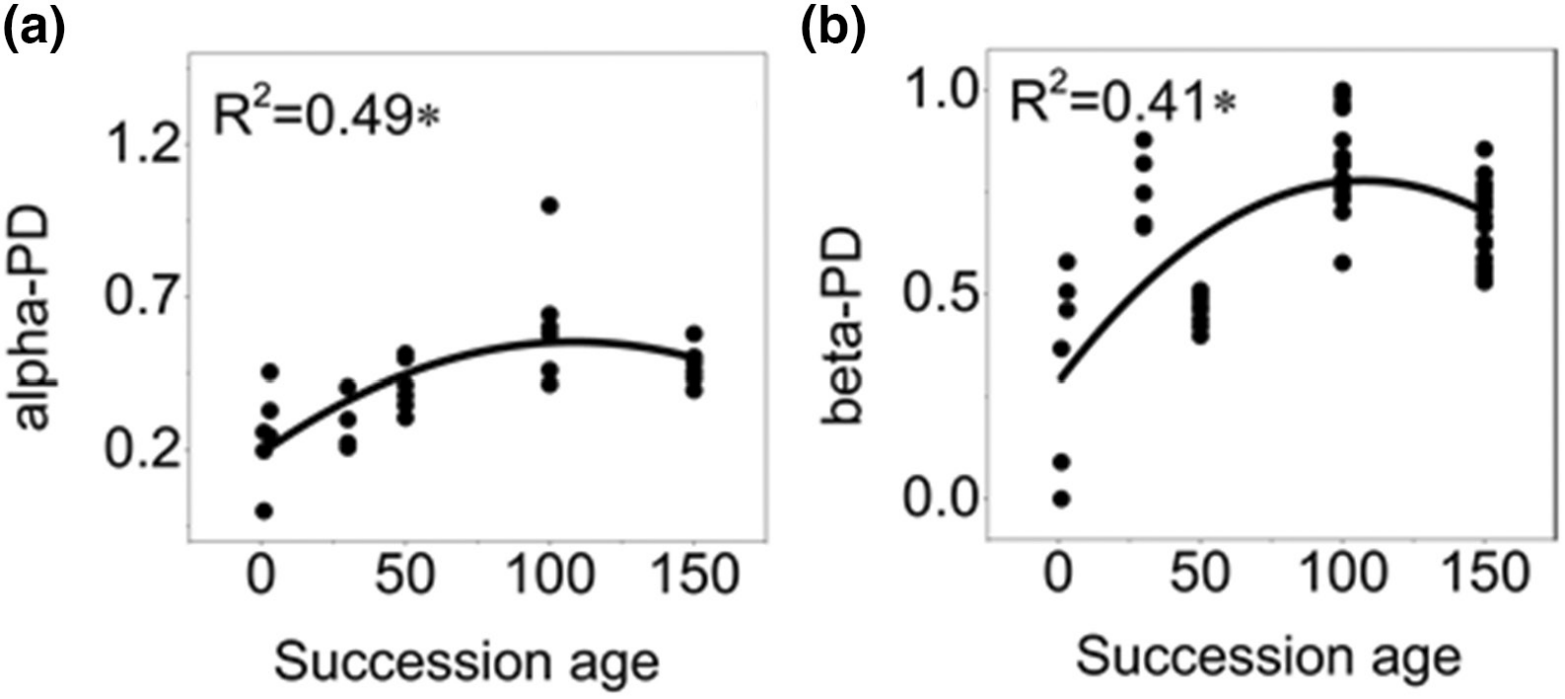
Quadratic polynomial regression for phylogenetic alpha (a) and beta diversity (b) as dependent variable and successional age as the independent variable.

**FIGURE 5:**
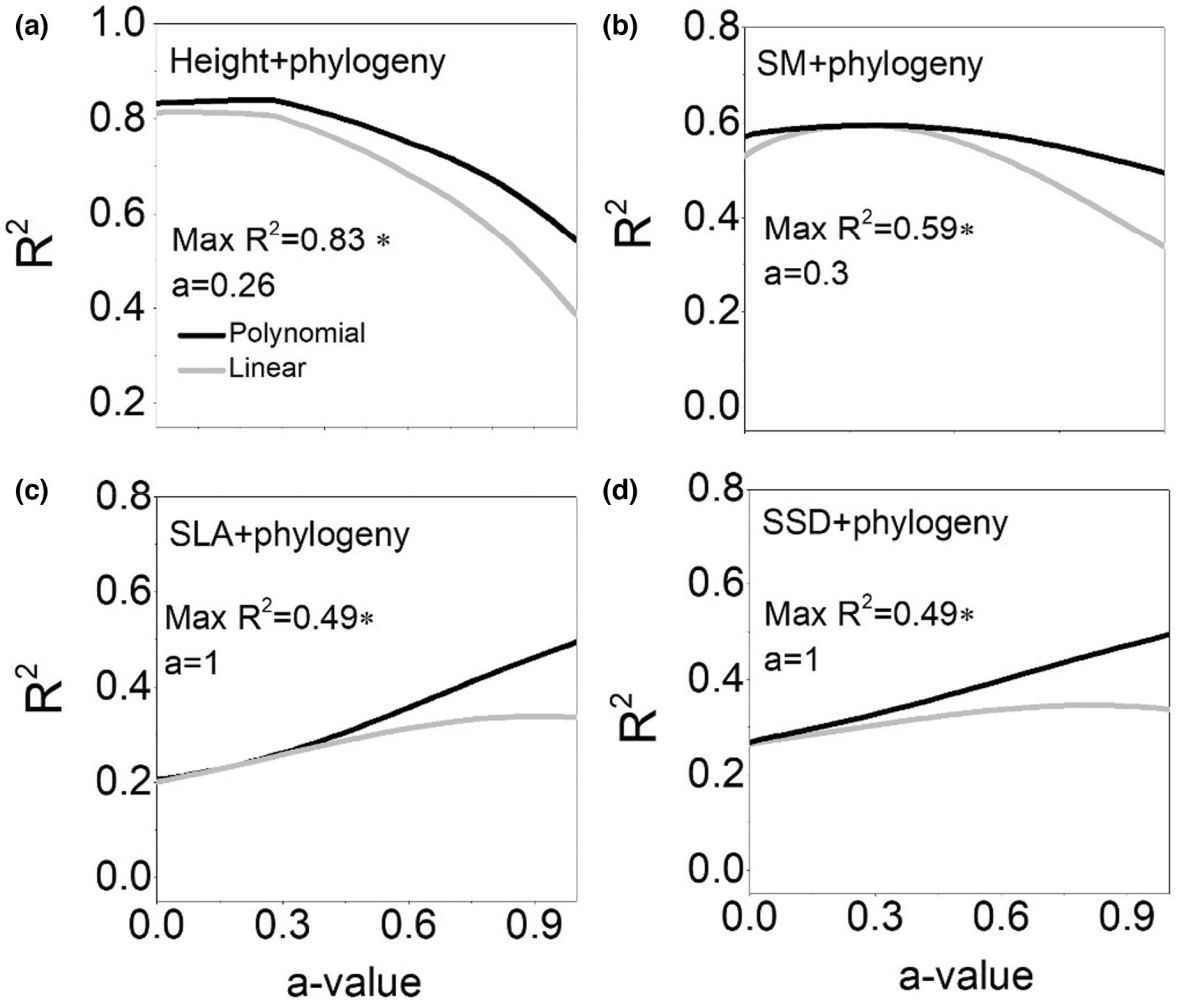
Integrating traits and phylogeny to maximize the *R* ^2^ values of the regression between alpha FPD and succession age across the range of a‐values (a–d). The value of *a*‐value ranges from 0 which only trait information is taken into account to 1 which only phylogenetic information is considered, in steps of 0.01. At intermediary *a*‐values, both trait distance and phylogenetic distance are combined. Height, plant height; SLA, specific leaf area; SM, seed mass; SSD, stem‐specific density.

**FIGURE 6:**
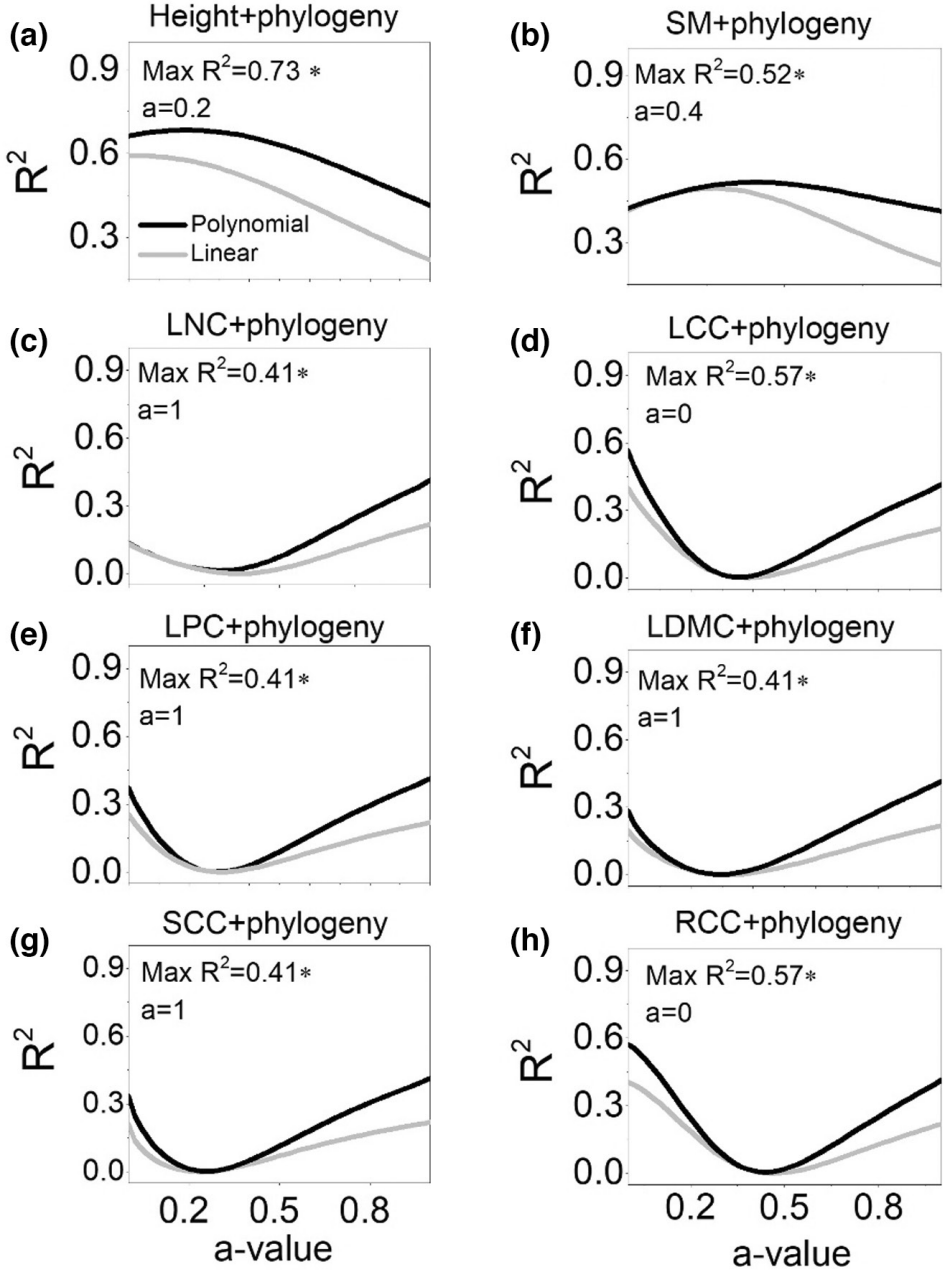
Integrating traits and phylogeny to maximize the *R*‐squared values of the regression between beta FPD and succession age across the range of *a*‐values (a–h). The value of *a*‐value ranges from 0 where only trait information is taken into account to 1 where only phylogenetic information is considered, in steps of 0.01. At intermediary *a*‐values, both trait distance and phylogenetic distance are combined. Height, plant height; LCC, leaf carbon content; LDMC, leaf dry mass content; LNC, leaf nitrogen content; LPC, leaf phosphorus content; RCC, root carbon content; SCC, stem carbon content; SM, seed mass.

Both phylogenetic alpha diversity and beta diversity increased firstly with succession ages and then declined at the last stage, indicating more or less incongruent pattern with functional diversity (Figure 4). Integrating phylogenetic information with height and SM can enhance the goodness of fit (*R* ^2^) compared with considering traits or phylogenetic information solely either for alpha and beta diversity (Figures 5 and 6). However, integrating other traits with phylogenetic distances did not show this advantage (Figures 5 and 6).

## 4 DISCUSSION

### 4.1 Functional diversity is structured by different traits and trait components at different scales

Our results support the hypothesis that functional diversity is structured by different traits at different scales. SSD and SLA are more appropriate in assessing alpha diversity compared with beta diversity, indicating their characteristic scale dependence. SSD and SLA are related to species competition for water and light (Roderick & Berry, 2001). Loess Plateau is a typical semi‐arid region in China, and the limitation of water and light increased at the advanced stages of forest secondary succession along with species recruitment (Wang et al., 2008). Therefore, differentiation of SSD and SLA within community promotes the species coexistence on the Loess Plateau, and this effect increases with successional process. The functional beta diversity is structured by six stoichiometric traits (LCC, RCC, SCC, LDMC, LPC, LNC) that contributed little to alpha diversity, suggesting that interspecific variation in plant nutrient concentrations is a major factor driving species turnover. Previous research has demonstrated the importance of leaf stoichiometry for plant nutrient utilization efficiency and its relationship with soil nutrients (Yan et al., 2016). In our study, functional beta diversity based on the six stoichiometric traits is more or less related to the heterogeneity of soil condition among plots, especially for soil rapid available phosphorus (PO_4_‐P) (Figure S5). Therefore, progressively similar soil condition may promote the convergence of plant stoichiometric traits among communities at later successional stages.

However, height and SM were meaningful in assessing both alpha and beta diversity. Height and SM are associated with resource capture, dispersal ability, and light competition (Laughlin et al., 2010). They were sensitive to either biotic interactions within community or environmental filtering among communities. For secondary forest succession, dominant species at later successional stages generally have higher plant height and bigger SM than that at early successional stages (Wilfahrt et al., 2014), at the same time niche differentiation of plant height and SM within community at later successional community was more significant than that at early community due to increasing competition or invasion of distant‐related species (Li et al., 2015). Therefore, plant height and SM are not only related to species coexistence within community (alpha diversity) but also to species replacement among communities (beta diversity) during forest succession.

According to the trait–gradient analysis, alpha trait components are the difference between a species trait values and the mean of co‐occurring species; beta trait components refer to a species position along a gradient defined by community‐level mean trait values (Ackerly & Cornwell, 2007). Interestingly, we observed that alpha trait components were more appropriate for alpha diversity analyses, and that beta trait components were more appropriate for beta diversity analyses. Previous studies on community diversity over forest succession did not relate alpha and beta traits values to different scales and thus could not disentangle the interaction among plant traits because of scale–trait mismatch (Purschke et al., 2013; Thorn et al., 2016). We used the concepts of alpha and beta traits to decompose traits into different components that are logically associated with different community assembly processes (Ackerly & Cornwell, 2007). Generally, species turnover among communities is caused by their macrohabitat preferences or environmental tolerances, whereas species coexistence within communities is mainly determined by traits involved in niche differentiation resulting from resource exploitation, microhabitat use, or other factors (Ackerly et al., 2006). Therefore, the trait components sensitive to alpha‐scale processes contributed little to diversity patterns among communities, whereas the trait components strongly associated with species turnover among communities were not appropriate for analysis of alpha diversity. Our results emphasize the importance of matching traits with scale in studying community functional diversity during forest succession.

### 4.2 Phylogenetic and functional diversity showed distinct patterns during succession

In successional theory, at the alpha scale, the biotic interaction (e.g., competition) plays a more important role in shaping community composition later in succession and leads to the increase of functional and phylogenetic diversity simultaneously (Lohbeck et al., 2012; Purschke et al., 2013; Zirbel et al., 2017). However, a contrasting pattern between phylogenetic and functional diversity was also found during a long‐term arable‐to‐grassland succession (Purschke et al., 2013). In our study, both phylogenetic alpha diversity and beta diversity increased firstly and then declined at the climax stages, which is more or less different from the monotonic increasing of alpha functional diversity or increasing/declining beta functional diversity. In fact, increasing competition does not necessary lead to increasing phylogenetic divergence (Bennett et al., 2013), and a severe or disturbed environment may be likely to lead to phylogenetic clustering (Helmus et al., 2010; Letten et al., 2014). Due to Loess Plateau's semi‐arid climate, serious water and soil erosion are major factors limiting plant growth over succession (Chai et al., 2015; Wang et al., 2018), and more species in later succession stage may increase the limiting effect of water condition along succession. Thus, colonists at the advanced successional stage generally are phylogenetically more similar to the residents during the recruitment process, which leads to the *Quercus* species dominating climax forest communities. This contributes to the decline of phylogenetic diversity at the climax communities (Figure S6). However, functional differentiation of key traits (e.g., height SM, SLA, and SSD, closely related to resource acquisition) within community is necessary for stable coexistence of closely related species at advanced successional stages. This is also consistent with a finding that species within genera can show a high trait variability if they occupy a particular environments position along environmental gradient (Hermant et al., 2012).

### 4.3 Integrating more conserved traits with the phylogenetic information enhance the ability to detect diversity pattern during succession

Integration of trait and phylogenetic information is increasingly used to gain novel insights into the driving mechanism of community assembly and diversity changes (Gianuca et al., 2016; Li et al., 2015; Sena et al., 2018). The underlying assumption is that integrating phylogenetic data with trait‐based analyses can enrich the test of community assembly or diversity hypotheses (Mouquet et al., 2012; Spasojevic et al., 2014). In our study, we did observe that combination of phylogenetic distance and the height and SM distance increased the *R* ^2^ either in alpha or beta diversity analyses. SM and plant height have the strongest phylogenetic signal across all spatial scales. Moreover, they were the only two traits that explain both alpha and beta trait variations across forest succession. However, combining phylogenetic with other traits reduced the overall explanatory power compared with using traits or phylogeny at either alpha or beta scales. Therefore, phylogenetic dissimilarity indeed captures a part of the trait niche of species, but not all of its dimensions, which limits the informativeness of integration of traits and phylogeny. The environmental and biotic selection pressures may alter the strength of other traits and phylogeny correlation, thus the information provided by plant traits and phylogenetic distances is asymmetric.

### 4.4 An implication for study on community succession

As ecosystems worldwide are degraded by natural and anthropogenic disturbances, it is important to evaluate the extent to which secondary forests recover ecological functions in addition to species composition (Funk et al., 2008; Kissing & Powers, 2010). Plant functional traits provide a common currency that allows assessment of the generality of patterns and predictions for biodiversity changes during succession (McGill et al., 2006). However, it is impossible to measure all plant functional traits related to the distribution of species. Although integration of phylogenetic and trait data in diversity metrics may make up for this deficiency, their scale dependency should be considered in assessing diversity (Gianuca et al., 2016; Leibold et al., 2010; Li et al., 2017).

We demonstrate that different traits and trait components structured community functional diversity at different scales during forest secondary succession on the Loess Plateau. Interestingly, phylogenetic, and functional diversity showed distinct patterns during forest secondary succession, and phylogenetic dissimilarities did not capture all information of plant functional traits because of scale–traits match. We conclude that semi‐arid stressful environment on the Loess Plateau has altered the phylogenetic conservation of plant traits, leading contrasting variation of community functional and phylogenetic diversity during forest secondary succession. Therefore, considering the roles of scale–traits match and phylogeny in studying species coexistence and community diversity would enable more possibilities for successful and effective ecological restoration programs in disturbed ecosystem in the future.

## AUTHOR CONTRIBUTIONS

**Yongfu Chai:** Writing – original draft (lead). **Shen Qiu:** Resources (equal). **Kaiyue Wang:** Formal analysis (equal). **Jinshi Xu:** Data curation (equal). **Yaoxin Guo:** Writing – review and editing (equal). **Mao Wang:** Formal analysis (equal). **Ming Yue:** Writing – review and editing (equal). **Mingjie Wang:** Investigation (equal). **Jiangang Zhu:** Investigation (equal).

## FUNDING INFORMATION

The study was financially supported by the National Natural Science Foundation of China (31700348 and 41571500).

## CONFLICT OF INTEREST STATEMENT

All of the undersigned authors participated actively in the study, and none has any potential conflict of interest. All of authors have read and approved the manuscript in its present form.

## Supporting information


Appendix S1

## Data Availability

The datasets supporting the conclusions of this article will be archived in Figshare upon acceptance. DOI: 10.6084/m9.figshare.20408112.
