# Supplementary material for: Partitioning and integrating of plant traits and phylogeny in assessing diversity along secondary forest succession in Loess Plateau of China
Source: Ecol Evol. 2023 May 10;13(5):e10055. doi: 10.1002/ece3.10055 (PMC10170657; doi:10.1002/ece3.10055)
Supplement: Supplementary file 1 — Appendix S1 [file ECE3-13-e10055-s001.docx]

Appendix 1

**Table S1** List of the measured traits, abbreviations, units and ecological strategy

| Group | Traits | Abbr. | Units | Strategy |  |
| --- | --- | --- | --- | --- | --- |
| Leaf traits | leaf nitrogen content | LNC | mg/g | Resource capture |  |
|  | leaf carbon content | LCC | mg/g | Resource capture and defence |  |
|  | leaf phosphorus content | LPC | mg/g | Resource capture |  |
|  | leaf N:P ratio | L N:P | ratio | Resource capture |  |
|  | leaf dry mass content | LDMC | mg/g | Leaf structure/water balance |  |
|  | specific leaf area | SLA | m^2^/kg | Resource capture |  |
| Stem traits | stem nitrogen content | SNC | mg/g | Resource capture |  |
|  | stem carbon content | SCC | mg/g | Resource capture and defence |  |
|  | stem specific density | SSD | g/cm^3^ | Stem structure |  |
|  | stem dry mass content | SDMC | mg/g | Stem structure/water balance |  |
| Root traits | root nitrogen content | RNC | mg/g | Resource capture |  |
|  | root carbon content | RCC | mg/g | Resource capture and defence |  |
|  | root dry mass content | RDMC | mg/g | Root structure/water balance |  |
| Seed trait | Seed Mass | SM | g per 1000 seeds | Dispersal ability |  |
| Plant trait | Plant Height | Height | m | Light competition |  |

**Table S2** List of the 210 species in the study

| **Code** | **Family** | **Genus** | **Species** |
| --- | --- | --- | --- |
| 1 | Araliaceae | Acanthopanax | *Acanthopanax_setchuenensis* |
| 2 | Sapindaceae | Acer | *Acer_ginnala* |
| 3 | Sapindaceae | Acer | *Acer_grosseri* |
| 4 | Sapindaceae | Acer | *Acer_oliverianum* |
| 5 | Sapindaceae | Acer | *Acer_pictum* |
| 6 | Ranunculaceae | Aconitum | *Aconitum_carmichaeli* |
| 7 | Ranunculaceae | Actaea | *Actaea_asiatica* |
| 8 | Campanulaceae | Adenophora | *Adenophora_polyantha* |
| 9 | Campanulaceae | Adenophora | *Adenophora_stricta* |
| 10 | Rosaceae | Agrimonia | *Agrimonia_pilosa* |
| 11 | Poaceae | Agropyron | *Agropyron_cristatum* |
| 12 | Simaroubaceae | Ailanthus | *Ailanthus_altissima* |
| 13 | Amaranthaceae | Amaranthus | *Amaranthus_retroflexus* |
| 14 | Vitaceae | Ampelopsis | *Ampelopsis_bodinieri* |
| 15 | Fabaceae | Amphicarpaea | *Amphicarpaea_trisperma* |
| 16 | Ranunculaceae | Anemone | *Anemone_vitifolia* |
| 17 | Ranunculaceae | Aquilegia | *Aquilegia_oxysepala_yabeana* |
| 18 | Araceae | Arisaema | *Arisaema_erubescens* |
| 19 | Aristolochiaceae | Aristolochia | *Aristolochia_kaempferi_* |
| 20 | Asteraceae | Artemisia | *Artemisia_annua* |
| 21 | Asteraceae | Artemisia | *Artemisia_apiacea* |
| 22 | Asteraceae | Artemisia | *Artemisia_argyi* |
| 23 | Asteraceae | Artemisia | *Artemisia_capillaris* |
| 24 | Asteraceae | Artemisia | *Artemisia_deversa* |
| 25 | Asteraceae | Artemisia | *Artemisia_eriopoda* |
| 26 | Asteraceae | Artemisia | *Artemisia_gmelinii* |
| 27 | Asteraceae | Artemisia | *Artemisia_igniaria* |
| 28 | Asteraceae | Artemisia | *Artemisia_japonica* |
| 29 | Asteraceae | Artemisia | *Artemisia_mongolica* |
| 30 | Asteraceae | Artemisia | *Artemisia_subdigitata* |
| 31 | Poaceae | Arthraxon | *Arthraxon_hispidus* |
| 32 | Liliaceae | Asparagus | *Asparagus_cochinchinensis* |
| 33 | Asteraceae | Aster | *Aster_ageratoides* |
| 34 | Asteraceae | Atractylodes | *Atractylodes_lancea* |
| 35 | Betulaceae | Betula | *Betula_platyphylla* |
| 36 | Gesneriaceae | Boea | *Boea_hygrometrica* |
| 37 | Poaceae | Bothriochloa | *Bothriochloa_ischaemum* |
| 38 | Poaceae | Bromus | *Bromus_japonicus* |
| 39 | Apiaceae | Bupleurum | *Bupleurum_chinense* |
| 40 | Poaceae | Calamagrostis | *Calamagrostis_pseudophragmites* |
| 41 | Fabaceae | Campylotropis | *Campylotropis_macrocarpa* |
| 42 | Cyperaceae | Carex | *Carex_breviaristata* |
| 43 | Cyperaceae | Carex | *Carex_lanceolata* |
| 44 | Asteraceae | Carpesium | *Carpesium_cernuum* |
| 45 | Betulaceae | Carpinus | *Carpinus_turczaninowii* |
| 46 | Vitaceae | Cayratia | *Cayratia_japonica* |
| 47 | Celastraceae | Celastrus | *Celastrus_hypoleucus* |
| 48 | Rosaceae | Cerasus | *Cerasus_clarofolia* |
| 49 | Rosaceae | Cerasus | *Cerasus_tomentosa* |
| 50 | Amaranthaceae | Chenopodium | *Chenopodium_glaucum* |
| 51 | Ranunculaceae | Cimicifuga | *Cimicifuga_foetida* |
| 52 | Asteraceae | Cirsium | *Cirsium_setosum* |
| 53 | Poaceae | Cleistogenes | *Cleistogenes_serotina* |
| 54 | Ranunculaceae | Clematis | *Clematis_shensiensis* |
| 55 | Verbenaceae | Clerodendrum | *Clerodendrum_trichotomum* |
| 56 | Cornaceae | Cornus | *Cornus_controversa* |
| 57 | Cornaceae | Cornus | *Cornus_walteri* |
| 58 | Papaveraceae | Corydalis | *Corydalis_edulis* |
| 59 | Anacardiaceae | Cotinus | *Cotinus_coggygria* |
| 60 | Rosaceae | Cotoneaster | *Cotoneaster_acutifolius* |
| 61 | Rosaceae | Cotoneaster | *Cotoneaster_multiflorus* |
| 62 | Rosaceae | Cotoneaster | *Cotoneaster_zabelii* |
| 63 | Rosaceae | Crataegus | *Crataegus_hupehensis* |
| 64 | Poaceae | Cymbopogon | *Cymbopogon_distans* |
| 65 | Apocynaceae | Cynanchum | *Cynanchum_atratum* |
| 66 | Apocynaceae | Cynanchum | *Cynanchum_auriculatum* |
| 67 | Apocynaceae | Cynanchum | *Cynanchum_chinense* |
| 68 | Ranunculaceae | Delphinium | *Delphinium_grandiflorum* |
| 69 | Asteraceae | Dendranthema | *Dendranthema_indicum* |
| 70 | Poaceae | Deyeuxia | *Deyeuxia_arundinacea* |
| 71 | Caryophyllaceae | Dianthus | *Dianthus_chinensis* |
| 72 | Poaceae | Digitaria | *Digitaria_sanguinalis* |
| 73 | Dioscoreaceae | Dioscorea | *Dioscorea_nipponica* |
| 74 | Caprifoliaceae | Dipsacus | *Dipsacus_asperoides* |
| 75 | Elaeagnaceae | Elaeagnus | *Elaeagnus_pungens* |
| 76 | Elaeagnaceae | Elaeagnus | *Elaeagnus_umbellata* |
| 77 | Lamiaceae | Elsholtzia | *Elsholtzia_ciliata* |
| 78 | Lamiaceae | Elsholtzia | *Elsholtzia_stauntoni* |
| 79 | Berberidaceae | Epimedium | *Epimedium_brevicornu* |
| 80 | Orchidaceae | Epipactis | *Epipactis_helleborine* |
| 81 | Geraniaceae | Erodium | *Erodium_stephanianum* |
| 82 | Celastraceae | Euonymus | *Euonymus_alatus* |
| 83 | Celastraceae | Euonymus | *Euonymus_phellomanes* |
| 84 | Oleaceae | Forsythia | *Forsythia_suspensa* |
| 85 | Oleaceae | Fraxinus | *Fraxinus_chinensis* |
| 86 | Rubiaceae | Galium | *Galium_boreale* |
| 87 | Geraniaceae | Geranium | *Geranium_sibiricum* |
| 88 | Geraniaceae | Geranium | *Geranium_wilfordii* |
| 89 | Asteraceae | Gerbera | *Gerbera_anandria* |
| 90 | Rosaceae | Geum | *Geum_aleppicum* |
| 91 | Lamiaceae | Glechoma | *Glechoma_longituba* |
| 92 | Malvaceae | Grewia | *Grewia_biloba* |
| 93 | Fabaceae | Gueldenstaedtia | *Gueldenstaedtia_verna* |
| 94 | Liliaceae | Hemerocallis | *Hemerocallis_flava* |
| 95 | Asteraceae | Heteropappus | *Heteropappus_altaicus* |
| 96 | Cannabaceae | Humulus | *Humulus_scandens* |
| 97 | Saxifragaceae | Hydrangea | *Hydrangea_bretschneideri* |
| 98 | Clusiaceae | Hypericum | *Hypericum_monogynum* |
| 99 | Fabaceae | Indigofera | *Indigofera_amblyantha* |
| 100 | Asteraceae | Ixeris | *Ixeris_sonchifolia* |
| 101 | Oleaceae | Jasminum | *Jasminum_giraldii* |
| 102 | Juglandaceae | Juglans | *Juglans_cathayensis* |
| 103 | Juglandaceae | Juglans | *Juglans_mandshurica* |
| 104 | Asteraceae | Kalimeris | *Kalimeris_indica* |
| 105 | Lamiaceae | Lagopsis | *Lagopsis_supina* |
| 106 | Fabaceae | Lathyrus | *Lathyrus_quinquenervius* |
| 107 | Asteraceae | Leontopodium | *Leontopodium_japonicum* |
| 108 | Rubiaceae | Leptodermis | *Leptodermis_oblonga* |
| 109 | Fabaceae | Lespedeza | *Lespedeza_bicolor* |
| 110 | Fabaceae | Lespedeza | *Lespedeza_cuneata* |
| 111 | Fabaceae | Lespedeza | *Lespedeza_davurica* |
| 112 | Liliaceae | Lilium | *Lilium_tenuifolium* |
| 113 | Caprifoliaceae | Lonicera | *Lonicera_ferdinandii* |
| 114 | Caprifoliaceae | Lonicera | *Lonicera_fragrantissima* |
| 115 | Caprifoliaceae | Lonicera | *Lonicera_japonica* |
| 116 | Caprifoliaceae | Lonicera | *Lonicera_serreana* |
| 117 | Caprifoliaceae | Lonicera | *Lonicera_tangutica* |
| 118 | Rosaceae | Malus | *Malus_spectabilis* |
| 119 | Poaceae | Melica | *Melica_scabrosa* |
| 120 | Menispermaceae | Menispermum | *Menispermum_dauricum* |
| 121 | Lamiaceae | Mentha | *Mentha_haplocalyx* |
| 122 | Poaceae | Miscanthus | *Miscanthus sacchariflorus* |
| 123 | Moraceae | Morus | *Morus_alba* |
| 124 | Moraceae | Morus | *Morus_australis* |
| 125 | Lamiaceae | Nepeta | *Nepeta_cataria* |
| 126 | Betulaceae | Ostryopsis | *Ostryopsis_davidiana* |
| 127 | Oxalidaceae | Oxalis | *Oxalis_corniculata* |
| 128 | Fabaceae | Oxytropis | *Oxytropis_bicolor* |
| 129 | Caprifoliaceae | Patrinia | *Patrinia_heterophylla* |
| 130 | Lamiaceae | Perilla | *Perilla_frutescens* |
| 131 | Apocynaceae | Periploca | *Periploca_sepium* |
| 132 | Saxifragaceae | Philadelphus | *Philadelphus_incanus* |
| 133 | Orobanchaceae | Phtheirospermum | *Phtheirospermum_japonicum* |
| 134 | Phytolaccaceae | Phytolacca | *Phytolacca_acinosa* |
| 135 | Araceae | Pinellia | *Pinellia_ternata* |
| 136 | Pinaceae | Pinus | *Pinus_bungeana* |
| 137 | Pinaceae | Pinus | *Pinus_tabulaeformis* |
| 138 | Anacardiaceae | Pistacia | *Pistacia_chinensis* |
| 139 | Plantaginaceae | Plantago | *Plantago_asiatica* |
| 140 | Cupressaceae | Platycladus | *Platycladus_orientalis* |
| 141 | Poaceae | Poa | *Poa_annua* |
| 142 | Poaceae | Poa | *Poa_nemoralis* |
| 143 | Liliaceae | Polygonatum | *Polygonatum_cirrhifolium* |
| 144 | Liliaceae | Polygonatum | *Polygonatum_odoratum* |
| 145 | Polygonaceae | Polygonum | *Polygonum_multiflorum* |
| 146 | Polygonaceae | Polygonum | *Polygonum_runcinatum* |
| 147 | Rosaceae | Potentilla | *Potentilla_chinensis* |
| 148 | Rosaceae | Potentilla | *Potentilla_paradoxa* |
| 149 | Rosaceae | Potentilla | *Potentilla_recta* |
| 150 | Rosaceae | Prunus | *Prunus_davidiana* |
| 151 | Rosaceae | Pyrus | *Pyrus_betulifolia* |
| 152 | Fagaceae | Quercus | *Quercus_aliena* |
| 153 | Fagaceae | Quercus | *Quercus_dentata* |
| 154 | Fagaceae | Quercus | *Quercus_variabilis* |
| 155 | Fagaceae | Quercus | *Quercus_wutaishanica* |
| 156 | Lamiaceae | Rabdosia | *Rabdosia_japonica* |
| 157 | Plantaginaceae | Rehmannia | *Rehmannia_glutinosa* |
| 158 | Rhamnaceae | Rhamnus | *Rhamnus_davurica* |
| 159 | Rhamnaceae | Rhamnus | *Rhamnus_utilis* |
| 160 | Asteraceae | Rhaponticum | *Rhaponticum_uniflorum* |
| 161 | Crassulaceae | Rhodiola | *Rhodiola_henryi* |
| 162 | Saxifragaceae | Ribes | *Ribes_emodense* |
| 163 | Fabaceae | Robinia | *Robinia_pseudoacacia* |
| 164 | Rosaceae | Rosa | *Rosa_hugonis* |
| 165 | Rubiaceae | Rubia | *Rubia_ovatifolia* |
| 166 | Rosaceae | Rubus | *Rubus_corchorifolius* |
| 167 | Polygonaceae | Rumex | *Rumex_dentatus* |
| 168 | Rhamnaceae | Sageretia | *Sageretia_perpusilla* |
| 169 | Adoxaceae | Sambucus | *Sambucus_williamsii* |
| 170 | Rosaceae | Sanguisorba | *Sanguisorba_officinalis* |
| 171 | Apiaceae | Saposhnikovia | *Saposhnikovia_divaricata* |
| 172 | Asteraceae | Saussurea | *Saussurea_japonica* |
| 173 | Schisandraceae | Schisandra | *Schisandra_chinensis* |
| 174 | Lamiaceae | Scutellaria | *Scutellaria_baicalensis* |
| 175 | Crassulaceae | Sedum | *Sedum_aizoon* |
| 176 | Poaceae | Setaria | *Setaria_viridis* |
| 177 | Asteraceae | Sinacalia | *Sinacalia_tangutica* |
| 178 | Liliaceae | Smilacina | *Smilacina_japonica* |
| 179 | Smilacaceae | Smilax | *Smilax_china* |
| 180 | Smilacaceae | Smilax | *Smilax_stans* |
| 181 | Asteraceae | Sonchus | *Sonchus_oleraceus* |
| 182 | Fabaceae | Sophora | *Sophora_davidii* |
| 183 | Rosaceae | Spiraea | *spiraea_japonica* |
| 184 | Rosaceae | Spiraea | *Spiraea_pubescens* |
| 185 | Rosaceae | Spiraea | *Spiraea_trilobata* |
| 186 | Poaceae | Spodiopogon | *Spodiopogon_sibiricus* |
| 187 | Lamiaceae | Stachys | *Stachys_sieboldi* |
| 188 | Caryophyllaceae | Stellaria | *Stellaria_media* |
| 189 | Poaceae | Stipa | *Stipa_capillata* |
| 190 | Cornaceae | Swida | *Swida_macrophylla* |
| 191 | Ranunculaceae | Thalictrum | *Thalictrum_przewalskii* |
| 192 | Malvaceae | Tilia | *Tilia_dictyoneura* |
| 193 | Anacardiaceae | Toxicodendron | *Toxicodendron_vernicifluum* |
| 194 | Liliaceae | Trillium | *Trillium_tschonoskii* |
| 195 | Ulmaceae | Ulmus | *Ulmus_parvifolia* |
| 196 | Urticaceae | Urtica | *Urtica_laetevirens* |
| 197 | Caprifoliaceae | Viburnum | *Viburnum_betulifolium* |
| 198 | Adoxaceae | Viburnum | *Viburnum_dilatatum* |
| 199 | Caprifoliaceae | Viburnum | *Viburnum_mongolicum* |
| 200 | Caprifoliaceae | Viburnum | *Viburnum_schensianum* |
| 201 | Fabaceae | Vicia | *Vicia_sepium* |
| 202 | Violaceae | Viola | *Viola_acuminata* |
| 203 | Violaceae | Viola | *Viola_japonica* |
| 204 | Violaceae | Viola | *Viola_phalacrocarpa* |
| 205 | Violaceae | Viola | *Viola_philippica* |
| 206 | Violaceae | Viola | *Viola_variegata* |
| 207 | Verbenaceae | Vitex | *Vitex_negundo* |
| 208 | Verbenaceae | Vitex | *Vitex_negundo_L._*var*._heterophylla* |
| 209 | Vitaceae | Vitis | *Vitis_piasezkii* |
| 210 | Vitaceae | Vitis | *Vitis_vinifera* |

**Table S3.** Functional alpha and beta diversity are structured by different traits during succession. Significant relationships (R^2^) were selected as meaningful traits at a certain scale. Functional diversity was calculated based on single trait and multiple traits respectively. The explanations of trait acronyms can be found in Table S1.

| **Traits** | **alpha-FD** | | **beta-FD** | | |  |
| --- | --- | --- | --- | --- | --- | --- |
|  | **R** | **R^2^** | | **R** | **R^2^** | |
| **Height** | **0.87** | **0.76** | | **0.77** | **0.59** | |
| **SM** | **0.70** | **0.50** | | **0.59** | **0.35** | |
| **SSD** | **0.38** | **0.14** | | 0.10 | 0.01 | |
| **SLA** | **0.37** | **0.14** | | 0.17 | 0.03 | |
| **SCC** | -0.16 | 0.03 | | **-0.38** | **0.14** | |
| **RCC** | -0.20 | 0.04 | | **-0.58** | **0.33** | |
| **LPC** | -0.10 | 0.01 | | **-0.36** | **0.13** | |
| **LDMC** | 0.04 | 0.00 | | **-0.34** | **0.12** | |
| **LCC** | -0.08 | 0.01 | | **-0.56** | **0.31** | |
| **LNC** | 0.31 | 0.09 | | **-0.35** | **0.12** | |
| **RDMC** | -0.10 | 0.01 | | -0.08 | 0.01 | |
| **SDMC** | 0.25 | 0.06 | | 0.31 | 0.09 | |
| **RNC** | 0.07 | 0.01 | | 0.12 | 0.02 | |
| **N:P** | 0.34 | 0.12 | | -0.06 | 0.00 | |
| **SNC** | -0.13 | 0.02 | | -0.22 | 0.05 | |
| **All traits** | **0.61** | **0.37** | | 0.12 | 0.02 | |

Significant results (P<0.05) are given in bold.


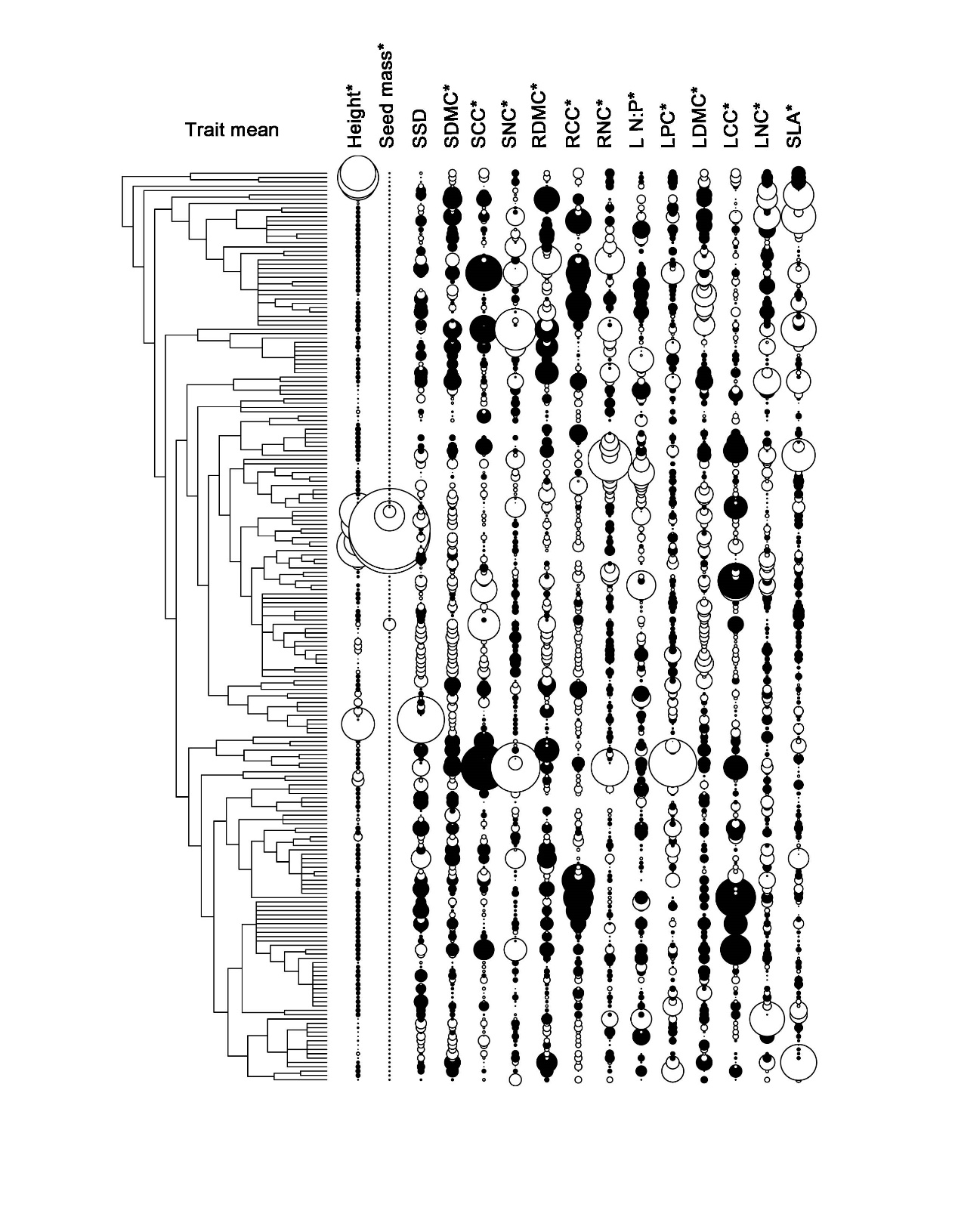


**Figure S1** Phylogeny of the 210 species. Species mean trait of the fifteen functional traits are represented on the tips of the phylogeny (in centred and standardised format). Traits with a signiﬁcant phylogenetic signal are represented by an *. The explanations of trait acronyms can be found in Table S1.

**
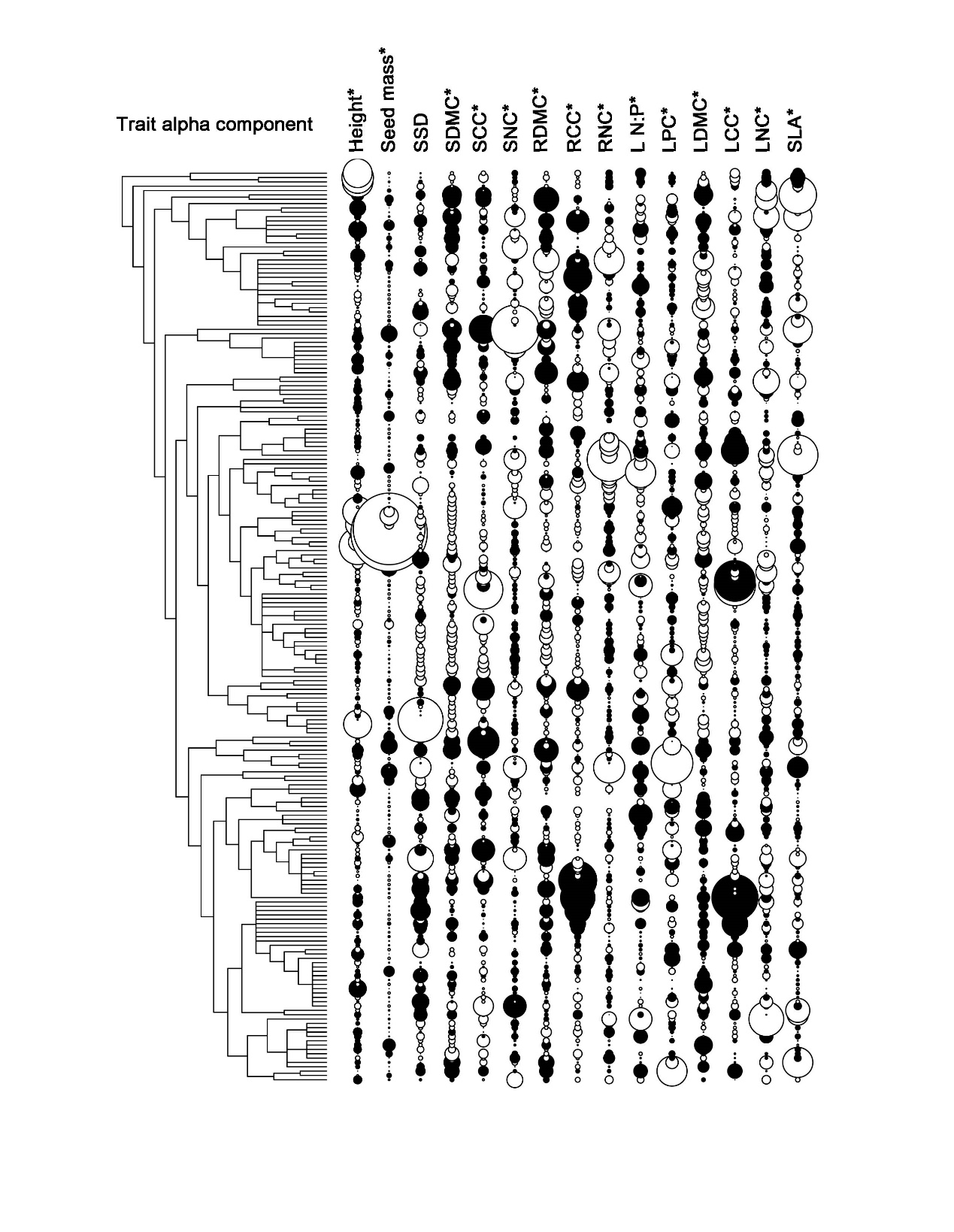
**

**Figure S2** Phylogeny of the 210 species. Trait alpha components of the fifteen functional traits are represented on the tips of the phylogeny (in centred and standardised format). Traits with a signiﬁcant phylogenetic signal are represented by an *. The explanations of trait acronyms can be found in Table S1.

**
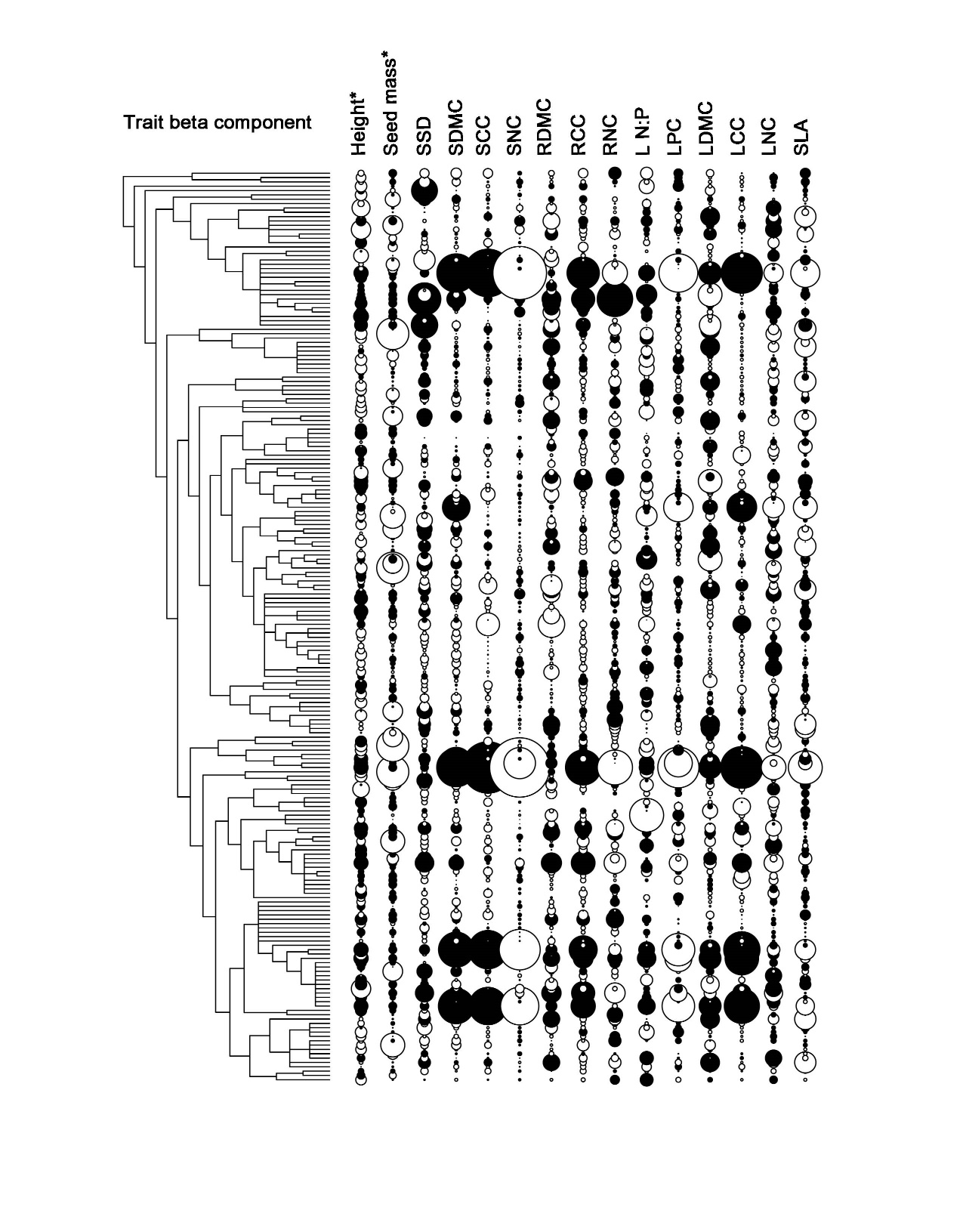
**

**Figure S3** Phylogeny of the 210 species. Trait beta components of the fifteen functional traits are represented on the tips of the phylogeny (in centred and standardised format). Traits with a signiﬁcant phylogenetic signal are represented by an *. The explanations of trait acronyms can be found in Table S1.


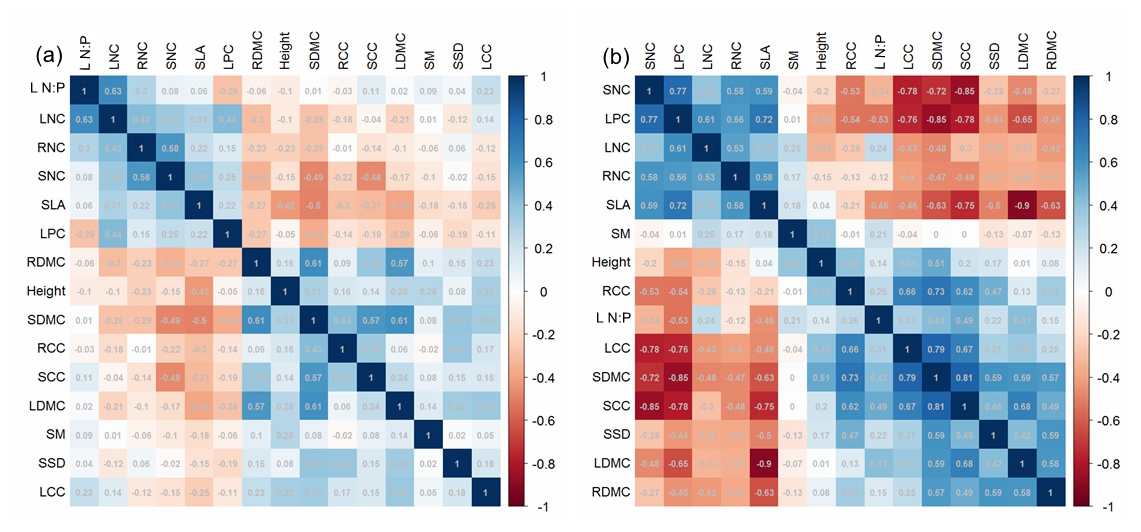


**Figure S4** Correlations between traits, (a) trait alpha components and (b) trait beta components. Blue represents positive correlation; Red represents negative correlation; Darkness of the color represents significance. The explanations of trait acronyms can be found in Table S1.


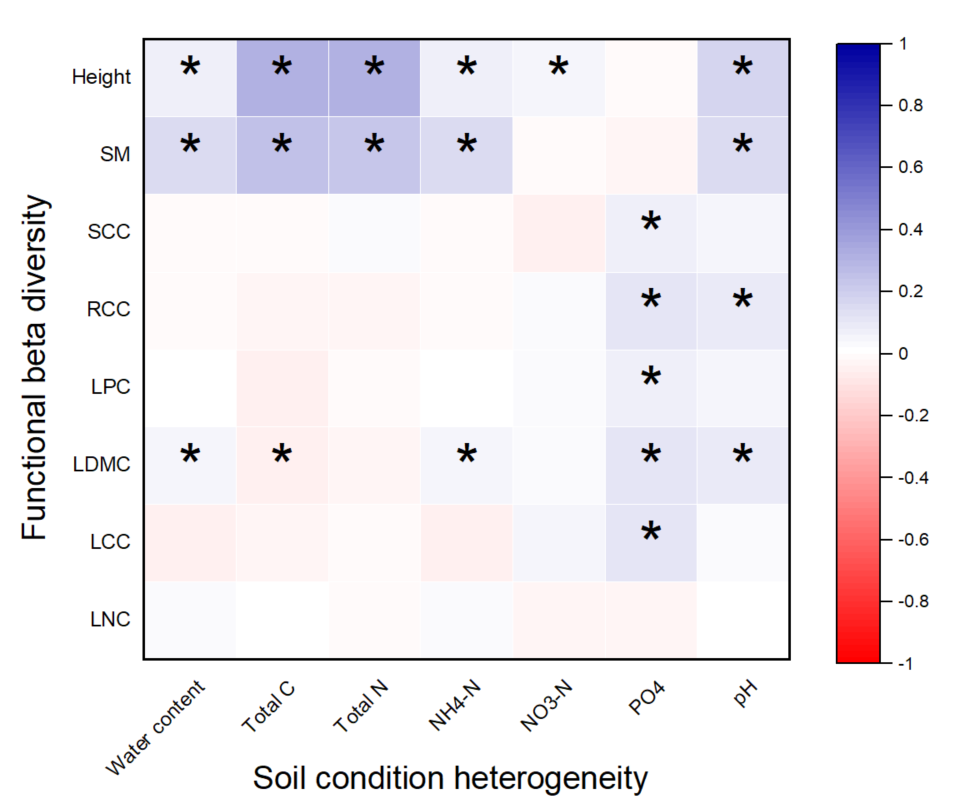


**Figure S5** Correlations between functional beta diversity and soil condition heterogeneity. Soil condition heterogeneity was assessed by Euclidean distance of soil factors between two plots. Blue represents positive correlation; Red represents negative correlation; Asterisk represents significant correlations. Soil data is from Chai et al. 2019. The explanations of trait acronyms can be found in Table S1.


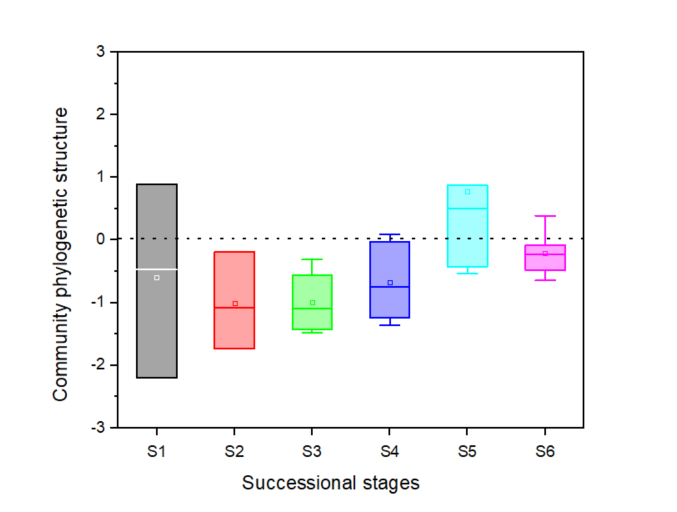


**Figure S6** Community phylogenetic structure of different successional stages. Community phylogenetic structure index was calculated by the function ‘ses.mpd’ in the Picante statistical package for R software. Positive structure index indicates phylogenetic overdispersion, negative structure index indicates phylogenetic clustering.
